# Attendance at Cervical Cancer Screening and Use of Diagnostic and Therapeutic Procedures on the Uterine Cervix Assessed from Individual Health Insurance Data (Belgium, 2002-2006)

**DOI:** 10.1371/journal.pone.0092615

**Published:** 2014-04-01

**Authors:** Marc Arbyn, Valérie Fabri, Marleen Temmerman, Cindy Simoens

**Affiliations:** 1 Unit of Cancer Epidemiology, Scientific Institute of Public Health, Brussels, Belgium; 2 Intermutualistic Agency (IMA/AIM), Brussels, Belgium; 3 Department of Gynaecology and Obstetrics, Ghent University, Ghent, Belgium; 4 Director, Reproductive Health and Research, World Health Organization, Geneva, Switzerland; 5 Laboratory of Cell Biology and Histology, University of Antwerp, Antwerp, Belgium; University of Missouri Kansas CIty School of Medicine, United States of America

## Abstract

**Objective:**

To assess the coverage for cervical cancer screening as well as the use of cervical cytology, colposcopy and other diagnostic and therapeutic interventions on the uterine cervix in Belgium, using individual health insurance data.

**Methods:**

The Intermutualistic Agency compiled a database containing 14 million records from reimbursement claims for Pap smears, colposcopies, cervical biopsies and surgery, performed between 2002 and 2006. Cervical cancer screening coverage was defined as the proportion of women aged 25–64 that had a Pap smear within the last 3 years.

**Results:**

Cervical cancer screening coverage was 61% at national level, for the target population of women between 25 and 64 years old, in the period 2004–2006. Differences between the 3 regions were small, but varied more substantially between provinces. Coverage was 70% for 25–34 year old women, 67% for those aged 35–39 years, and decreased to 44% in the age group of 60–64 years. The median screening interval was 13 months. The screening coverage varied substantially by social category: 40% and 64%, in women categorised as beneficiary or not-beneficiary of increased reimbursement from social insurance, respectively. In the 3-year period 2004–2006, 3.2 million screen tests were done in the target group consisting of 2.8 million women. However, only 1.7 million women got one or more smears and 1.1 million women had no smears, corresponding to an average of 1.88 smears per woman in three years of time. Colposcopy was excessively used (number of Pap smears over colposcopies = 3.2). The proportion of women with a history of conisation or hysterectomy, before the age of 65, was 7% and 19%, respectively.

**Conclusion:**

The screening coverage increased slightly from 59% in 2000 to 61% in 2006. The screening intensity remained at a high level, and the number of cytological examinations was theoretically sufficient to cover more than the whole target population.

## Introduction

For the year 2010, 593 new cases of cervical cancer (World-age standardised rate (W-ASR) 7.5/100,000 women-years) were reported by the Belgian Cancer Registry [www.kankerregister.org/], and the most recent estimates for 2008 showed that approximately 275 women (W-ASR 2.7/100,000 women-years) died from the disease [1], [2]. Age-period-cohort analyses revealed an increased risk of cervical cancer for cohorts born after 1940, that was counteracted partially by screening [3], [4]. Through well-organised cytological screening of high quality, the incidence of cervical cancer can be reduced substantially [5]–[10]. In Belgium, screening remained essentially opportunistic, which means that Pap smears are taken at the spontaneous initiative of the woman, her gynaecologist or her general practitioner [11], [12]. Opportunistic screening often results in a high level of overscreening and a heterogeneous quality[13]. The Belgian cervical cancer screening policy is adapted from European Guidelines and foresees one Pap smear or liquid-based cytology sample every three years for women of 25 to 64 years of age [14]–[16]. Nevertheless, the level of adherence to this policy is rather poor, also in the Flemish provinces where in the mid-1990s, a program was set up involving invitation of women in the target age range 25-64 [11], [12].

Optimal attendance of the target population is one of the main determinants of success of a screening program [17]. In the past, in Belgium, this attendance could only be assessed by surveys [18], [19]. Such surveys, involving collection of information directly from women, are known to suffer from selection and reporting biases that systematically result in overestimated coverage rates [20]–[22]. Recently, more reliable methods for estimating the population coverage have become available through the compilation of health insurance data by the Intermutualistic Agency (IMA). In a previous report, IMA data were used to assess the cytological screening coverage, as well as the consumption of medical acts related to collection and interpretation of Pap smears, and follow-up or treatment of women with cervical lesions, comprising the period 1996-2000. The current study completes the 1996-2000 report [12], allowing assessment of the cervical cancer screening activity in Belgium for more than a decade.

## Methods

Health insurance in Belgium is mandatory, covering the whole Belgian population, and is mediated by "sickness funds" that arrange reimbursements and keep track of all reimbursed medical acts [12]. Upon request of the Scientific Institute of Public Health (Brussels, Belgium), a data file containing more than 14 million individual patient reimbursement records was compiled by the Intermutualistic Agency (IMA). This data file contained all medical acts related to cervical screening and diagnostic or therapeutic interventions on the uterine cervix (Pap smear collection and interpretation, colposcopies, cervical biopsies and their interpretation, surgery on the cervix) performed on women resident in Belgium, between 2002 and 2006. The database incorporates medical acts performed in all types of services (private physicians, group practices, private and public outpatient clinics and hospitals), but does not contain diagnostic or clinical information.

A numerical individual ID code, age, date of the act, residence of the woman, and type of the medical act was provided in the data set. The ID code was a unique number, allowing tracing multiple consecutive Pap smears and other acts for the same woman. For the respect of privacy, data details were truncated to reduce the risk of obtaining cells of cross tables with small counts (<5). Age was converted into the respective five-year age group at the exception of the group 0–14 and the 75+ age group, which were each grouped into one category. The calendar date of the act was restricted to the year of the act at the exception of the collection of cervical cell specimen, where also the month was available. The residence was restricted to the province. Contrary to the previous analysis of the period 1996–2000, the social status was provided, whereas the geographical detail of district was restricted to province. Social status was categorised as following: beneficiary of increased reimbursement (BIR), normal status, unknown or censored status. Increased reimbursement is foreseen for vulnerable social categories such as orphans, widows, aged or retired people, and individuals being unemployed for long delays, with a handicap and low income.

### Screening in Belgium

Cervical cancer screening during the study period was cytology-based [11], [12]. In spite of clinical guidelines proposing one Pap smear every three years for women aged 25–64 years, no restriction was imposed regarding reimbursement. Invitations were sent to women of the target population in last halve of the 1990s in all five provinces of Flanders (Northern Belgium), but this was continued during the study period in only two of them (Antwerp and Flemish-Brabant).

Colposcopy was recommended in case of a first observation of high-grade squamous intraepithelial lesion (HSIL), atypical glandular cells (AGC) or HPV-positive ASC-US, or after a second observation of atypical squamous cell of undermined significance (ASC-US) or low-grade squamous intraepithelial lesions (LSIL) [23], [24]. The prevalence of these lesions, estimated from a comprehensive provincial cervical cytology registry was: 0.4% for HSIL, 1.1% for LSIL, 2.2% for ASCUS and 0.1% for atypical glandular cells [25]. Professional guidelines were in place regarding diagnostic and therapeutic work-up [26].

### Statistical analyses

Data were aggregated by age groups and geographical levels and combined with the respective mid-period female population size, obtained from the Directorate General Statistics and Economic Information (DGSEI, formerly known as the National Institute of Statistics, Brussels, Belgium) to compute proportions or incidence rates. The following geographical levels were distinguished: the whole country, the three regions (Flemish Region, Capital Region of Brussels, and the Walloon Region) and the eleven provinces.

Cervical cancer screening coverage was defined as the proportion of the target population (women of 25–64 years) that had a cervical cytology examination within the last 3 years. Overuse (or excess use) was defined as the proportion of cervical cytology specimen taken in the target group that did not contribute to the coverage (number of smears taken in 3-years time/number of women screened in that period – 1)*100.

For the computation of the cumulative incidence of conisation or hysterectomy until a given age *k*, the following formula was applied:
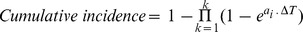



where Π stands for cumulative product, *a_i_* for age-specific incidence, and Δ*T* for the amplitude of the age categories [27].

## Results

### Screening coverage

The 3-year cervical cancer screening coverage in the target population (25-64y), assessed over the period 2004-06, was 61%. Similar to the preceding analysis (1996–2000), the range of variation in screening coverage at the level of the Regions was small: 60% in the Flemish Region, 62% in the Brussels-Capital Region and 63% in the Walloon Region. The variation in coverage, measured as an absolute difference in proportions, over the period 2002–2006, was very limited (+0.5% for the whole country), with a small increase in the Flemish (+0.8%) and Walloon Region (+0.5%) and a decrease in the Brussels-Capital Region (–0.4%) (Table 1). Differences at provincial level were more substantial, with a screening coverage, observed in 2006, ranging from 51% (Luxembourg) to 70% (Walloon-Brabant).

**Table 1:** Consumption of Pap smears, three-year screening coverage and overuse for women between 25 and 64 year old, by Region (Belgium, 1996–2006).

Region	Period	Mean female population (25–64 years)	Number of smears taken	Number of women screened <3years ago		#smears/ #women ratio	Excess use
					3-year coverage		
Flemish Region	1996–1998	1,582,128	1,569,687	870,520	55.0%	0.99	80.3%
	1998–2000	1,587,847	1,691,932	911,761	57.4%	1.07	85.6%
	2002–2004	1,600,240	1,742,417	947,756	59.2%	1.09	84.0%
	2004–2006	1,614,285	1,778,783	968,385	60.0%	1.10	84.0%
Brussels-Capital Region	1996–1998	253,557	264,611	134,397	53.0%	1.04	96.9%
	1998–2000	255,656	292,582	147,381	57.6%	1.14	98.5%
	2002–2004	267,190	328,664	166,451	62.3%	1.23	97.0%
	2004–2006	274,052	330,150	169,654	61.9%	1.20	95.0%
Walloon Region	1996–1998	867,361	944,176	506,947	58.4%	1.09	86.2%
	1998–2000	873,429	1,010,866	532,113	60.9%	1.16	90.0%
	2002–2004	887,683	1,069,667	557,633	62.8%	1.21	92.0%
	2004–2006	897,182	1,091,051	568,004	63.3%	1.22	92.0%
Whole of Belgium	1996–1998	2,703,047	2,778,474	1,511,864	55.9%	1.03	83.8%
	1998–2000	2,716,933	2,995,380	1,591,255	58.6%	1.10	88.2%
	2002–2004	2,755,113	3,140,748	1,671,840	60.7%	1.14	87.9%
	2004–2006	2,785,516	3,199,984	1,706,043	61.2%	1.15	87.6%

Overall, the screening coverage increased with 2.2% compared to 2000. This increase was more pronounced in the Brussels-Capital Region (+4.3%) and between 2 and 3% for the other two regions. In most provinces, the screening coverage increased (between 0.9% and 4.3%), at the exception of 2 provinces where a small decrease was noted (Limburg (–0.2%) and Luxemburg (–0.3%)).

### Age groups

**Figure 1:**
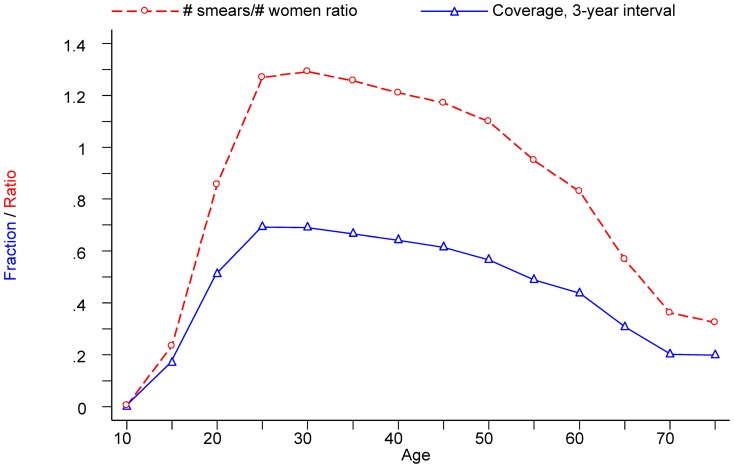
Variation of the screening coverage and the # smears/ # women ratio considered over a 3-year interval, by 5-year age group (Belgium, 2004-06).

At the national level, the youngest age groups (women of 25–34 years old), were the best screened with a coverage of 70%. From the age of 35 to 49, the coverage decreased gradually from 67% to 62%. From the age of 50, the coverage dropped more steeply to reach 44% in the age group 60–64 (Figure 1). The age profile was similar in the three regions. However, in the Brussels-Capital Region and Walloon Region the decline in the age group 50–64 was less pronounced than in the Flemish Region.

### Screening interval and screening beyond the target age range

The median screening interval was 13 months (inter-quartile range: 11–22 months). A time span of 36 months or more was observed in only 3% of women with 2 or more smears in the studied time period, whereas in 30% it was less than 12 months.

**Figure 2:**
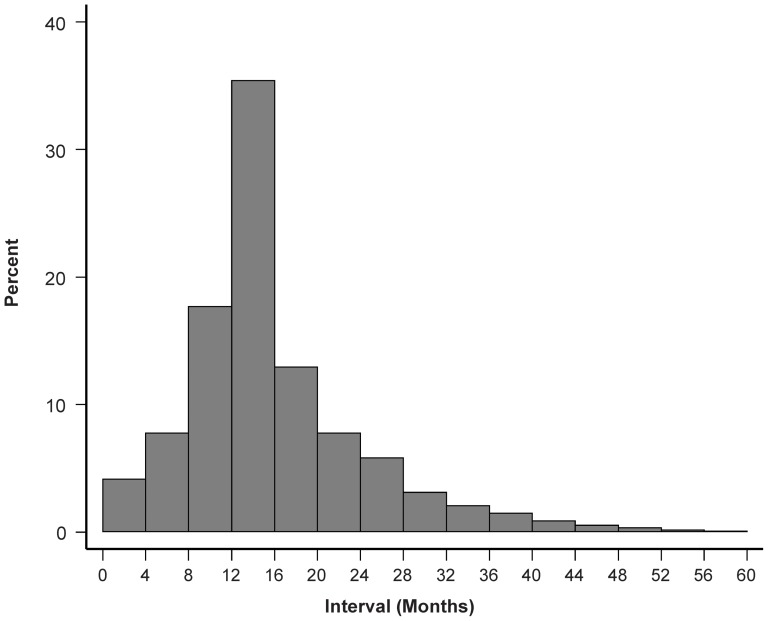
Distribution of the time interval between successive collections of cervical cytology specimen.

Screening beyond the target age range contributed 18% of all smears: 10% from women younger than 25 years and 8% from women aged 65 or older. In the age groups 15–19 and 20–25, the screening coverage was 17% and 52%, respectively. A coverage of 31% and 21% was noted in the older age groups 65–69 and 70–74, respectively (see Figure 2).

### Social status

**Figure 3:**
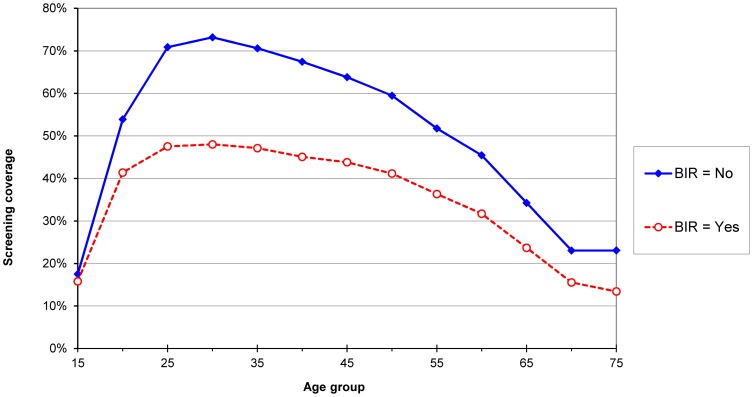
Three-year screening coverage by age and by socio-economic status, defined as beneficiary or not beneficiary of increased reimbursement (BIR), Belgium, 2004 –**2006.**

In the whole Belgian target population, the screening coverage was respectively 40% in women who benefited from increased reimbursement for health care (BIR) compared to 64% for women who did not benefit from increased reimbursement (non-BIR). The differences between the two social categories (non-BIR - BIR) were consistent over all geographical areas and all groups, but varied in magnitude. At regional level, the difference varied between 21% (Brussels-Capital Region) and 27% (Flemish Region). At provincial level, the difference ranged between 21% (Brussels) and 33% (Walloon-Brabant). The contrast changed also by age group: in the range 22–25% for women aged 25–44 years and less for younger and older women (Figure 3).

### Consumption of Pap smears

For the period 2004–2006, the ratio of the number of Pap smears over the size of the target population was 1.15. In absolute figures: 3.2 million Pap smears were interpreted in the period 2004–2006 which were taken from only 1.7 million women. One million and seventy nine thousand women, accounting for 39% of the target group, did not get a Pap smear in this three-year period.

The excess use of cervical cytological examinations was 88%, which means that each screened women received on average 1.88 smears over a time span of three years. The excess smear use was high in all parts of Belgium. However, it was less high in the Flemish Region (84%), highest in the Capital Region (95%), and intermediate in the Walloon Region (92%) (Table 1).

### Profession of smear takers

Eighty nine percent of cervical cell specimens were collected by gynaecologists. The proportion of Pap smears prepared by general practitioners (GPs) was low and varied substantially by Region. In a decade (1996–2006), the contribution of GPs decreased continuously: from 26% to 14% in the Flemish Region, from 10% to 7% in Brussels and from 5% to 2% in the Walloon Region.

### Colposcopy use

One colposcopic examination was charged for every 3.2 Pap smears, at the national level. This ratio varied regionally between 7.8 (Flemish Region) and 1.6 (Walloon Region) (Table 2). At provincial level, the lowest Pap smear/colposcopy ratio was observed in Hainaut and Liège (1.6) and the highest in Antwerp (11.4).

**Table 2:** Number of colposcopies, cervical biopsies, and Pap smears; ratio of the number of biopsies over the number of colposcopies, ratio of the number of Pap smears over the number of colposcopies, and ratio of the number of biopsies over the number of Pap smears, Belgium, 2002–2006.

				Biopsy/	Pap/	Biopsy/
			Pap	colposcopy	colposcopy	Pap
Region	Colposcopies	Biopsies	smears	ratio	ratio	ratio
Flemish Region	441,027	48,559	3,456,854	0.110	7.8	0.014
Brussels-Capital Region	229,164	12,460	677,749	0.054	3.0	0.018
Walloon Region	1,292,537	30,654	2,230,035	0.024	1.7	0.014
Whole of Belgium	1,980,927	93,773	6,417,936	0.047	3.2	0.015

The biopsy/colposcopy ratio was low (on average 5%), due to the very high frequency of not-clinically indicated colposcopies (Table 2).

### Conisations

**Figure 4:**
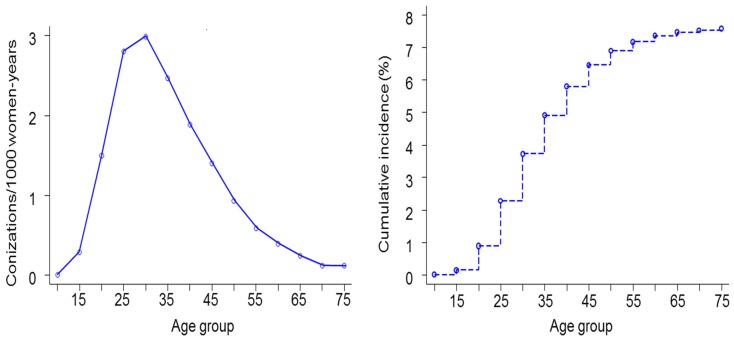
Incidence of conisation by age (left); cumulative incidence of conisation up to a given age (right) in Belgium, 2002 –**2006.**

The age-specific incidence of excisional treatment for cervical precancer (by conisation or large loop excision of the transformation zone) and the cumulative incidence of treatment up to a given age is shown in Figure 4. Only the first conisations were taken into account. The incidence of conisation peaks in the age group 30–34 years (3.0/1000 women-years). Up to the age of 34 years, 3.7% of women have had a history of conisation. Up to the age of 65, this proportion was 7.3%.

### Hysterectomies

**Figure 5:**
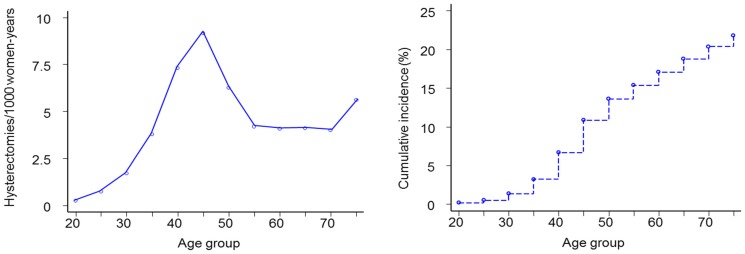
Incidence of total hysterectomy by age (left); cumulative incidence of total hysterectomy up to a given age (right) in Belgium, 2002 –**2006.**

The incidence of hysterectomy was highest in the age range 40–54 years: 7.3, 9.2, and 6.3 per 1000 women-years in age groups 40–44, 45–49, and 50–54 years, respectively (Figure 5-left). Up to the age of 49 years, 14.8% of women have their uterus, including their cervix, removed (Figure 5-right). Up to the age of 64, this proportion was 18.7%.

The shape of the curves was similar for the three regions. However, the age-specific incidences were substantially higher in the Flemish and Walloon Regions, compared to the Brussels-Capital Region. Peak incidences, in the 45–49 year age group were 9.4, 9.0, and 7.4 per 1000 women-years in the Flemish, Walloon and Brussels-Capital Region, respectively.

Nineteen percent of women of 20 years or older, hysterectomised in 2002, had at least one Pap smear taken in the subsequent three years.

## Discussion

### Screening coverage, intensity of screening

This report (2002–2006) shows results similar to those of the previous analysis (1996–2000) [12]. In 2006, at the national level, 61% of women 25–64 years old had received a Pap smear in the last 3 years, what is only 2% higher compared to 2000. The screening coverage was substantially lower among socially vulnerable groups: 40% among women with BIR, whereas 64% among women without BIR.

The slightly higher coverage in the Walloon Region compared to the Flemish Region observed in 1996–2000 was maintained, which is remarkable, given the fact that in the latter region a screening campaign was organised since the mid-1990s. The intensity of the invitations was relaxed in the Flemish provinces after 2000: it was maintained in Antwerp and Flemish-Brabant, and completely interrupted in East-Flanders. No obvious impact of sending invitations to women was seen. For, instance: the increase in screening coverage observed between 2000 and 2006 was +2.6% in East-Flanders and only +1.8% in Flemish-Brabant.

Also the excess use of Pap tests hardly changed over time. This overuse has a substantial impact on the public health budget without clear benefit in terms of incidence or mortality reduction compared to a more conservative screening as recommend in European guidelines [28]–[30].

### Colposcopies

An impressive amount of colposcopies are performed in Belgium. Although the main purpose of colposcopy is to assist in the diagnosis of precancer in women with a previous cytological abnormality, it is often performed at the same time of the collection of a screening Pap smear. Colposcopy was obviously not used according to accepted guidelines [23], [24] However, a small relative decrease was observed over time in the use of colposcopy. One colposcopy was performed per 2.9 Pap smears, in 2000, versus 3.2 Pap smears/colposcopy, in 2006.

### Conisations

Over-consumption of screen tests, inevitably generates unnecessary follow-up examinations (repeat Pap smears, colposcopies, biopsies) and treatment of lesions. Excisional treatment of cervical intraepithelial neoplasia (CIN), in particular deep excisions beyond 10 mm of depth, may be associated with an increased risk of preterm delivery [31]–[34,34]. Under the assumption that pregnant women previously treated with LLETZ exceeding 10 mm have a risk of preterm delivery (PD) that is at least 1.5 times higher than for non-treated pregnant women [31], [33]. Taking into account the maternal age distribution at delivery [35], and the cumulative incidence of conisation over age (figure 4-right), we can estimate that 1.5% of all PDs in Belgium are attributable to prior LLETZ. However, among treated women, the risk of PD attributable to deep LLETZ excisions may be about 33%.

The incidence of conisations increased in Belgium from 1.3/1000 women years in 2000 to 1.7/1000 in 2006.

### Hysterectomies

No further screening is required for women who have undergone a total hysterectomy for a non-oncological indication. However, 26% of women aged 25–64 years, hysterectomised in 2002, had at least one Pap smear taken in the next three years. From other sources, it was estimated that 13% of total hysterectomies in Belgium are performed for a malignant indication, so these women can be considered as still requiring cytological testing. Unfortunately, the data provided by IMA do not provide the indication of the hysterectomy. In a survey in the United States, it was noted that 64% of hysterectomised women were still cytologically screened [36]. In Belgium, over-screening in women without cervix is substantially lower.

The incidence of hysterectomy decreased slightly over time (5.0 and 4.8 per 1000 women-years aged 25–64 years, in 2000 and 2006, respectively).

### Strengths and limitations of the study

Data details were truncated to reduce the risk of identifiability. This truncation impeded computation of indicators at district or municipality level.

No data were obtained for 2001 and the ID coding was different between the two study periods (1996–2000 and 2002–2006), impeding computation over more than five years.

The received IMA data contained only administrative content and were not linkable with medical registries. It must be remarked that organised population-based screening, includes by definition registration of individual data with the possibility of linkage between population-, screening-, cancer- and mortality-registers. The Belgian privacy protection law in principle does not allow registration of personal medical data but includes derogations specifically for population screening [37]. Moreover, since 2010, all cytopathology laboratories in Belgium are mandated, by law, to communicate all cyto-pathology results related to cancer screening to the National Cancer Registry [38]. So in the future, more in-depth evaluation of screening indicators will be possible, as recommended in European guidelines [16].

A major strength of the study comes from the exhaustivity of the IMA database allowing more reliable evaluation of the screening coverage than surveys. The national health interview surveys, conducted in 2004, reported a coverage of 72%, which was 12% higher than the estimate derived from the IMA database for that year [19], [28]. This discrepancy is probably due to reporting biases, which are inherent to interview surveys resulting in inflated coverage estimates [39]–[41].

### Structural propositions for the future

Measures foreseen in the European Council Recommendation on Cancer screening should be binding and applied in all the Belgian regions. The fact that hardly any evolution in screening indicators was observed over the last ten years, demonstrates the necessity of a well-organised cervical cancer screening programme and clear information for the physicians and women. Health authorities of the Federal and Community Governments and representatives of the scientific societies should define as soon as possible a rational, evidence-based and cost-effective cervical cancer screening policy for Belgium. In the context of the actual opportunistic screening, an organised cervical cancer screening programme should deal with the questions linked with the two major problems identified in the current study: (1) How can the excess consumption of Pap smears among currently screened women be reduced? (2) How can the 39% of the target population that is currently not covered be reached and convinced to participate regularly at recommended intervals.

### How to decrease over-screening?

In the meanwhile, reimbursement conditions in Belgium have changed. Previously, Pap smears were reimbursed without any interval restrictions, but since May 2009, two different types of cervix cytology examinations were distinguished: a) screening (minimum interval of 2 years) and b) follow-up (maximum 2 per year) [42]. A small financial contribution was paid by the individual concerned. The implementation of this regulation has reduced dramatically the total volume of cervical cytology examinations performed: from 1.37 million in 2008 to 0.81 million in 2010, or a reduction of 41%. In March 2013, a new Royal Decree was published that restricted reimbursement of screening cytology to once every three years. This three-yearly Pap smear is now completely free of charge [43]. Matching reimbursement regulations to recommended screening intervals appears to have had a tremendous effect. How these new regulations influenced the screening coverage and the average Pap smears/screened woman ratio has to be assessed in the third IMA report on cervical cancer screening. It should be noted, however, that some amount of over-screening, not reimbursed but paid by the woman herself, may occur.

### How to increase screening coverage?

As mentioned above, the impact of sending invitation letters with a recommendation to have a Pap smear taken, probably has had only minor effects on screening coverage. A working hypothesis is that GPs can play a role in reaching women who do not attend at screening. In a telephone survey, conducted in the Flemish Region, screened women indicated their gynaecologist as preferred smear taker, whereas non-screened women would accept the proposal of a GP to have a Pap smear taken [18]. These data provide substance for the working hypothesis. However, structural measures must be taken to create conditions promoting proactive preventive care, including secondary prevention of cervical cancer, to be offered systematically by the GP to his/her patients. The limited proportion of Pap smear taken by GPs, in particular in the Walloon Region, indicate that mobilizing GPs in efforts to increase screening coverage is not an easy issue.

Offering self-sampling kits for HPV testing to non-screened women is another strategy, which can induce coverage increase [44]. However, this should be carefully tested in pilot projects before considering general introduction. Moreover, a strategy requires a well-organised environment to be successful.

## Conclusions

Individual health insurance data makes up an enormous resource for epidemiological research and program evaluation in general and, in particular allows a precise estimate of the population coverage and the consumption of related medical acts.

This second IMA report reveals substantial overconsumption of resources with limited health benefits. The national cervical cancer screening coverage just reached 61%, but the number of smears was sufficient to cover more than the whole target population. The coverage varied slightly between regions but more substantially between provinces. However, no obvious evidence of an impact of the invitational programs set up by Flemish provinces could be discerned. The excessive use of low-cost colposcopy is striking.

### Précis

The amount of Pap smears examined is sufficient to cover the whole target population, however 39% is not covered. Substantial overconsumption of colopscopies is noted.
